# Making the GRADE (GRading and Admissions Data for England)

**DOI:** 10.23889/ijpds.v8i2.2265

**Published:** 2023-09-14

**Authors:** Merlin Walter, Nadir Zanini

**Affiliations:** 1 Ofqual, Coventry, United Kingdom

## Objectives

To provide a data owner’s perspective into the journey to create GRADE, an Ofqual-DfE-UCAS linked administrative dataset, and to make it available for independent research. Moving from the ethical principles underpinning the sharing of data and through multiple strands of the project, we will draw lessons to inform best practice.

## Methods

Areas of research interest were used as drivers to define the content of GRADE. To ensure that research questions of relevance to both policy makers and scholars could be addressed, we established an advisory committee including key representatives of the statistical, research, and educational policy communities. Thanks to the collaboration with technical staff from participating organisations, the data was linked to ensure that students’ anonymised data could be tracked across different data sources. We then focused on supporting external research by producing metadata and supplementary documentation, as well as establishing independent funding provision for projects using the GRADE data.

## Results

The internal research conducted was helpful to inform policy development and to test the data. The GRADE data was created and made available to external researchers. A data sharing framework was published to describe, at high-level, the content of the data and its potential use, as well as to set out how to access this rich source of micro-data. Detailed data specifications have been made available, alongside a low-fidelity synthetic version of the data.

Numerous research projects based on the GRADE data are currently underway and preliminary findings are already being disseminated. More research is being planned, based on an upcoming refreshed version of the GRADE data. Significant funding has been made available for external researchers to conduct independent work.

## Conclusion

Creating, combining, and sharing administrative data is not an easy task. Engagement with researchers and the wider community is necessary from the beginning of the project. It is a considerable investment, with potentially high returns, but also significant costs, and the output produced must align with policy and research priorities.

